# Metabolomics in Atrial Fibrillation: Unlocking Novel Biomarkers and Pathways for Diagnosis, Prognosis, and Personalized Treatment

**DOI:** 10.3390/jcm14010034

**Published:** 2024-12-25

**Authors:** Justyna Rohun, Danuta Dudzik, Joanna Raczak-Gutknecht, Elżbieta Wabich, Krzysztof Młodziński, Michał J. Markuszewski, Ludmiła Daniłowicz-Szymanowicz

**Affiliations:** 1 Department of Cardiology and Electrotherapy, Faculty of Medicine, Medical University of Gdansk, 80-214 Gdańsk, Poland; justynarohun@gumed.edu.pl (J.R.); wabich.ela@gmail.com (E.W.); krzysztofmlodzinski@gumed.edu.pl (K.M.); 2 Department of Biopharmaceutics and Pharmacodynamics, Faculty of Pharmacy, Medical University of Gdansk, 80-416 Gdańsk, Poland; danuta.dudzik@gumed.edu.pl (D.D.); jrgutknecht@gumed.edu.pl (J.R.-G.); michal.markuszewski@gumed.edu.pl (M.J.M.)

**Keywords:** atrial fibrillation, metabolic phenotype, metabolic profiling, metabolite, biomarker, gut microbiota

## Abstract

Background/Objectives: Atrial fibrillation (AF) is the most frequent arrhythmia in the adult population associated with a high rate of severe consequences leading to significant morbidity and mortality worldwide. Therefore, its prompt recognition is of high clinical importance. AF detection often remains challenging due to unspecific symptoms and a lack of reliable biomarkers for its prediction. Herein, novel bioanalytical methodologies, such as metabolomics, offer new opportunities for a better understanding of the underlying pathological mechanisms of cardiovascular diseases, including AF. The metabolome, considered a complete set of small molecules present in the organism, directly reflects the current phenotype of the studied system and is highly sensitive to any changes, including arrhythmia’s onset. A growing body of evidence suggests that metabolite profiling has prognostic value in AF prediction, highlighting its potential role not only in early diagnosis but also in guiding therapeutic interventions. By identifying specific metabolites as a disease biomarker or recognising particular metabolomic pathways involved in the AF pathomechanisms, metabolomics could be of great clinical value for further clinical decision-making, risk stratification, and an individual personalised approach. The presented narrative review aims to summarise the current state of knowledge on metabolomics in AF with a special emphasis on its implications for clinical practice and personalised medicine.

## 1. Introduction

**Figure 1:**
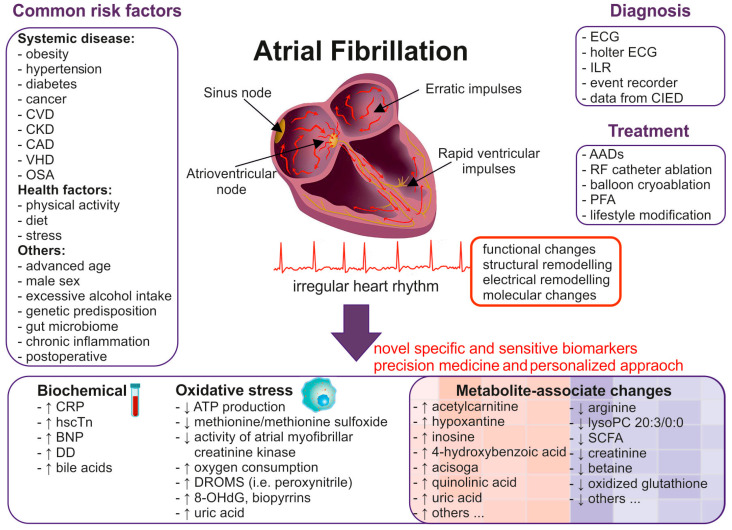
Atrial fibrillation overview. Various risk factors are presented, including metabolomic alterations, along with diagnostic and treatment methods. AADs—anti-arrhythmic drugs, ATP—adenosine triphosphate, CAD—coronary artery disease, CIED—cardiac implantable electronic device, CKD—chronic kidney disease, CRP—C-reactive protein, CVD—cardiovascular diseases, DD—D-dimer, DROMS—derivatives of reactive oxygen metabolites, ECG—electrocardiogram, hscTn—high-sensitivity cardiac troponin, ILR—implantable loop recorder, lysoPC—20:3lysophosphatidylcholine, OSA—obstructive sleep apnoea, PFA—pulse field ablation, 3RF—radiofrequency, SCFA—short-chain fatty acid, VHD—valvular heart disease, 8-OHdG—8-hydroxy-2′-deoxyguanosine.

Atrial fibrillation (AF) is the most common arrhythmia in the adult population [1,2]. According to the Global Burden of Disease 2019, over 59 million individuals are affected by AF [3]. As the prevalence is continuously rising [2] and increases with age [1,2,4], the disease is called a global epidemic [4], having severe medical and economic repercussions [2,5]. However, despite its frequency, AF often remains underdiagnosed due to its highly heterogenous symptomatology, which poses significant clinical challenges and frequently leads to severe consequences such as a heightened risk of thromboembolic events, including a 5-fold higher risk of stroke [6], exacerbations of heart failure (HF) [7], and even death (117,038 deaths in 2019) [8]. AF diagnosis is usually made either during medical work-up in symptomatic patients or, less frequently, accidentally on spontaneous electrocardiographic (ECG) records in asymptomatic individuals (Figure 1).

The mechanisms underlying AF are complex and still poorly understood [9,10]. Current theories include either circus movement in atria (micro-entry) or electrical hyperactivity from an ectopic focus (ectopic pacemaker) [9,10]. Nevertheless, all of the pro-arrhythmic mechanisms promoting AF share common features, including channelopathies, autonomic dysregulation, and structural atrial remodelling [11], ultimately resulting in atrial cardiomyopathy [12]. The severity of the arrhythmogenic substrate is crucial for predicting AF outcomes, particularly regarding arrhythmia management and anticoagulation, making a comprehensive understanding of AF pathophysiology essential for clinical practice. Interestingly, electrical and contractile remodelling, potentially driven by calcium current downregulation, is reversible after the restoration of sinus rhythm (SR) [13]. Therefore, finding specific and sensitive tools for AF prediction would be of particular interest, as available therapy methods are limited (Figure 1), rather symptomatic and may have possible side effects, and extensive costs are constantly being allocated for arrhythmia-driven complications and AF screening in multiple countries. However, none of the screening tools has been successful so far [2].

Multiple risk factors contribute to AF onset, including advanced age, male sex, obesity, excessive alcohol consumption, physical inactivity, obstructive sleep apnoea, hypertension, or valvular heart disease [1,2], but their predictive value remains limited [14]. Similarly, numerous laboratory findings, such as elevated serum levels of bile acids [15], cardiac high-sensitivity troponin (hsTn), brain natriuretic peptide (BNP), C-reactive protein, or D-dimer, have been linked to AF onset [16,17,18,19,20]. However, none of the identified factors has been proven sufficiently sensitive or specific for predicting AF onset, as their levels can rise in various other conditions. A growing body of evidence highlights the role of genetic predisposition in AF occurrence, including variations in genes related to ion channel function, structural proteins, and atrial fibrosis; however, the subject of its clinical importance is still under debate [1,2,14]. Therefore, to improve AF prevention and early recognition, new approaches and new, both specific and sensitive biomarkers are indispensable. Figure 1 depicts the functional mechanisms involved in AF pathogenesis, indications of available diagnostics, and treatment options.

Metabolomic analyses have been considered a promising tool in cardiovascular research [21]. As an integral part of system biology, metabolomics focuses on metabolites—the end products of cellular processes which directly reflect an organism’s specific chemical phenotype [22]. Changes in the metabolome occur before clinical symptoms and reflect dynamic perturbations in the genome, transcriptome, and proteome. Therefore, metabolomic analysis holds potential for elucidating disease mechanisms and predicting conditions such as AF. The method could be particularly beneficial in the management of patients with asymptomatic AF. A recent meta-analysis including 217,850 patients with AF showed them to have a similarly high risk of poor prognosis as symptomatic individuals [23]. Additionally, lower odds of receiving antiarrhythmic drugs or ablation therapy were observed, and, consequently, a higher risk of progression to the permanent form of the arrhythmia with its further consequences [23]. Therefore, metabolomics could be used for an early risk stratification of these patients and therapy guidance, as the issue remains unclear in current guidelines.

This narrative review aims to summarize the current state of metabolomics-based research in atrial fibrillation, with a particular focus on its clinical implications, therapeutic targets, and precision medicine. Furthermore, we explore how metabolomic findings can be translated into routine workflows, inform drug discovery, and enable patient stratification.

## 2. Metabolomic Insights to Understand Atrial Fibrillation

Metabolomics is a part of system biology and one of the ‘omics’ sciences, alongside genomics, proteomics, and transcriptomics [24], which allows for a comprehensive view of living organisms. It allows us to understand and predict complex physiological processes and observe how they are altered by disease or environmental factors. The metabolome refers to the collection of small molecules, typically under 1500 Da, known as metabolites, that participate in an organism’s biochemical pathways. These metabolites include endogenous compounds (such as amino acids, carbohydrates, lipids, nucleotides, and organic acids) produced as part of normal physiological processes, as well as exogenous compounds (such as pollutants, drugs, and food) [25,26]. State-of-the-art technologies like nuclear magnetic resonance (NMR) spectroscopy and mass spectrometry (MS), often combined with separation techniques like high-performance liquid chromatography (HPLC), have significantly advanced the ability to detect and analyse a wide range of metabolites [26,27,28]. However, one of the major challenges in metabolomics is managing the complexity and high dimensionality of data, with thousands of metabolites varying in chemical structure and abundance. Advanced feature selection techniques and dimensionality reduction methods are essential for extracting relevant biological information from these large datasets [29]. As the downstream expression of other ’omics’ layers, metabolomics directly reflects the organism’s current state, ongoing biological processes, and environmental interactions, thereby representing the phenotype at a specific moment [30,31]. Given this, metabolomics holds great potential for the early prediction of AF, as it provides a detailed snapshot of the organism’s metabolic profile and can help define an AF-specific metabotype. When integrated with other “omics” approaches such as genomics and proteomics and combined with molecular and clinical data, metabolomics can help uncover the biological processes and metabolic pathways involved in AF onset and progression. This aligns with the goals of precision medicine, where treatment is tailored based on a patient’s unique metabolic profile. By identifying metabolic signatures linked to AF, clinicians can make more accurate treatment decisions, reduce adverse effects, and improve therapeutic outcomes. Moreover, distinguishing between physiological and pathological pathways involved in AF-related metabolic processes can offer valuable insights for clinical decision-making, aiding in the prediction of arrhythmia onset and enabling preventive care for those at risk of AF.

### 2.1. Oxidative Stress Background in Atrial Fibrillation

Oxidative stress reflects an imbalance between oxidants and antioxidants, so the generated reactive oxygen species (ROS), which are unstable and highly reactive, and the capacity of the human body for detoxification or damage repair [32]. With the complex pathomechanism of AF, available evidence suggests that oxidative stress is a major mechanism in developing AF substrate that facilitates the occurrence and maintenance of AF [33]. Moreover, multiple risk factors for AF development, like heart failure (HF), diabetes mellitus (DM), or obesity, are also associated with an increase in oxidative stress, therefore linking oxidative stress with them and atrial arrhythmogenesis [34,35,36].

Reactive oxygen species are generated in the atria through electron leakage from mitochondria or the activation of enzymes (nicotinamide adenine dinucleotide phosphate oxidases, xanthine oxidase, and uncoupled nitric oxide synthase) [37]. These low molecular weight molecules are partially reduced derivatives of molecular oxygen and include superoxide, hydrogen peroxide, hydroxyl radicals, and peroxynitrite [37]. Under physiological conditions, low levels of ROS are indispensable for cellular signalling and homeostasis maintenance. Nevertheless, with the excess of ROS cellular levels and insufficient cell defence mechanisms to reduce excess free radicals (i.e., enzymes that directly metabolize ROS, such as catalase, superoxide dismutase, and glutathione peroxidase, or scavenging systems, including vitamin C or E, glutathione, and nicotinamide adenine dinucleotide), oxidative stress occurs [37]. ROS react with proteins, nucleic acids or lipids, causing aberrant enzyme function, DNA breakage or mutations or lipid peroxidation [37,38].

Four elements can be identified in the AF arrhythmogenic substrate: electrical remodelling, Ca^2+^ handling remodelling, structural remodelling, and the remodelling of the autonomic nervous system [39]. ROS target multiple redox-sensitive ion channels and Ca^2+^ handling proteins, modulate kinases which subsequently affect downstream molecules, or activate multiple signalling pathways (nuclear factor kappa B and transforming growth factor-β), and therefore enhance the pro-inflammatory and pro-fibrotic response in the atria [39].

Electrical remodelling occurs early and is partially reversible [39]. When ROS impair the function of several ionic currents (L-type Ca^2+^ current, inward-rectifier K^+^ current, ultra-rapid delayed rectifier K^+^ current, and the agonist-independent, constitutively active form of acetylcholine-dependent K+ current) or intercellular gap junctional communication, a shorter atrial effective refractory period (ERP) or action potential duration or altered activation and conduction, are seen, which promote re-entry [39,40]. Inappropriate ERP lengthening may also be observed, which is arrhythmogenic as well [39,40].

Regarding Ca^2+^ handling in atrial myocytes, ROS modulate ryanodine receptor type 2, sarcoplasmic reticulum calcium ATPase 2 A, and phospholamban function, therefore promoting AF [39,41].

ROS-driven structural atrial remodelling in AF is a late phenomenon and occurs secondary to prolonged exposure to risk factors [39]. It includes myocyte necrosis and apoptosis, myolysis, and interstitial fibrosis. The inhomogeneity of electrical conduction and interference of impulse propagation through atria is observed [42]. TGF-β1 and angiotensin II (ang II) signalling pathways seem to play a key role in the development of atrial fibrosis through myocyte hypertrophy and apoptosis, endothelial changes, fibroblast proliferation, collagen synthesis, and fibrosis [39].

Finally, the AF burden is increased through ROS-based autonomic nervous system modulation [39]. Evidence of both sympathetic and parasympathetic hyperinnervation is crucial in forming the AF substrate, and abnormal autonomic signalling (a thorough reduction of SOD1 expression) in the atria contributes to AF [39,43].

Available data indicate that oxidative stress is implicated in the pathophysiology of atrial remodelling, leading to atrial cardiomyopathy and, finally, AF [33,44,45,46]. Primarily, data provided by Mihm et al. suggest that peroxynitrite, found only in AF patients (compared to the control SR group), mediates oxidative cardiac damage [46]. Soulat-Dufour et al. speculated that there may be a correlation between the degree of oxidative stress in the fibrillatory atrial myocardium and the decreased methionine/methionine sulfoxide ratio found in the serum of patients with AF and low LA strain [47]. Biomarkers of oxidative DNA damage, such as 8-hydroxy-2′-deoxyguanosine (8-OHdG) or biopyrrin, were found to be higher in the serum of AF patients compared to the SR group in the general population [48]. In another study by Wu et al., urine F2-isoprostanes and isofurans at the end of cardiac surgery were significantly higher in post-operative AF individuals with levels of linear association with time [49]. Also, the activity of atrial myofibrillar creatinine kinase, a molecule highly sensitive to oxidative injury, is reduced in AF, leading to severe contractile dysfunction [46]. The levels of derivatives of ROS (DROM) in patients with persistent AF were significantly higher than in patients with paroxysmal AF [44]. Notably, after catheter ablation, patients with paroxysmal AF who were in the highest quartile of basal DROM levels showed a markedly increased recurrence rate of AF [44], which may serve as background for future research.

The pathophysiology of oxidative stress in AF is complex. A thorough understanding of the atrial sources of ROS, which vary with the duration and substrate of atrial fibrillation [50], the types of ROS that are involved, the triggers that cause ROS formation, and the downstream signalling cascades that result in arrhythmias, is necessary to develop an effective anti-oxidant treatment for AF [39]. Therefore, metabolomic analyses could help identify exact pathways and compounds responsible for oxidative stress in AF onset in particular patients, and further on, enable the development of antioxidant interventions targeting oxidative stress based on a personalized approach. Nevertheless, available data on targeting specific sources of ROS implicated in atrial remodelling is scarce and needs further research.

### 2.2. Uric Acid Is Linked to Oxidative Stress, Inflammation, and Atrial Fibrillation

Serum uric acid (SUA) is the final product of purine catabolism associated with oxidative damage and inflammation [51,52]. The molecule is produced by xanthine oxidase (XO), converting hypoxanthine to xanthine, and further on to uric acid (UA) and molecular oxygen as a side product. The basal expression of XO is low, but data proved that it can rise in various pro-inflammatory conditions [53]. Therefore, uric acid was proven as an independent factor for a high risk of cardiovascular mortality [54,55,56,57] and recently, has also been linked to the mechanisms behind AF [58,59,60,61,62,63,64,65,66]. Moreover, its elevated levels were demonstrated to positively correlate with AF risk factors, such as hypertension or metabolic syndrome [67].

Several theories concerning UA-induced AF have been proposed. Mechanisms include either the activation of XO, intracellular accumulation of soluble UA, or activation of the NLR family pyrin domain containing 3 (NLRP3) inflammasome induced by monosodium urate (MSU) crystals [52]. The final consequence is atrial structural and electrical remodelling [68].

XOR converts UA precursors into UA and can also be a source of reactive oxygen species (ROS), i.e., superoxide free radicals [51]. The increase in ROS exacerbates thrombosis, inflammation, and tissue remodelling as described previously in this review [37,39].

A growing body of evidence reports that intracellular UA accumulation leads to cellular damage by oxidative stress increase and the activation of the ERK1/2 pathway [69]. UA transporters (UAT) are involved in the intracellular accumulation of UA; therefore, its inhibition could be a potential treatment target for UA-driven AF [52].

Inflammation is one of the major factors in both initiating and perpetuating AF [20,70]. Elevated plasma levels of pro-inflammatory cytokines (interleukins and TNF-α) or CRP were repeatedly observed in AF patients [70]. An increase in SUA levels leads to MSU accumulation, which in turn activates the NLRP3 inflammasome [52]. In atrial cardiomyocytes, after the NLRP3 inflammasome complex activation, atrial electrical and structure remodelling occurs, with abnormal Ca^2+^ handling, ERP shortening, and atrial hypertrophy [52].

Various clinical studies connected AF with elevated SUA levels [62,63,64,65,66,71,72]. Based on a large (123,238 patients enrolled between 2006 and 2014) prospective cohort Chinese study reported by Li et al., it can be concluded that increased SUA level is associated with higher AF prevalence, as its levels were markedly higher in patients who developed new-onset AF (NOAF) [64]. The results were confirmed in a prospective, large-sample (15,382 participants) community-based American study [71], and a recent investigation of Swedish and Norwegian cohorts, where elevated SUA levels were independent risk factors for AF occurrence [62,65]. Of note, higher SUA levels in women with AF might imply some sex-specific mechanisms [66] and indicate the uricosuric oestrogen influence [72]. In a transgender man, plasma uric acid decreased in cases where adequate collections were obtained both before and throughout oestrogen therapy for this condition [72].

In a large American population-based prospective cohort study reported by Tamariz, including 15,382 AF-free black and white men and women, aged 45 to 64 years, from the Atherosclerosis Risk In Communities (ARIC) study, higher rates of AF were observed within the black race [71]. SUA levels were also increased in patients with ischemic HF [71] and in the group with new-onset AF after myocardial infarction [63]. Additionally, after cryoballoon-based catheter AF ablation, its higher levels were linked to more frequent recurrence episodes and therefore could be helpful for its prediction [73]. Similarly, high SUA levels could be useful for the prediction of NOAF after coronary artery bypass grafting procedures [58]. Therefore, uric acid may represent an easily determined AF prognostic biomarker. On the other hand, some other authors explain the connection between high SUA levels in AF and cardiovascular risk factor overload [74]. Due to the association of uric acid with other AF risk factors, like coronary artery disease, hypertension, diabetes, or metabolic syndrome, it cannot be perceived as an independent biomarker for its occurrence [74]. It was also proven that uric acid has a direct effect on smooth muscle cell proliferation [75], endothelial dysfunction, and decreased nitric oxide production [76], as well as on the local activation of the renin-angiotensin-aldosterone system( RAAS) [77]. The connection between the mechanism of AF and renin–angiotensin–aldosterone system (RAAS) activation is known, as with RAAS activation, atrial structural remodelling occurs [78,79]. Of note, the effects of several agents from the group of angiotensin receptor blockers or angiotensin-converting enzyme inhibitors on AF recurrence have been tested in experimental and clinical settings with variable results [80,81,82], indicating that the blockade of RAAS syndrome is not efficient in AF episode prevention. On the one hand, Pedersen et al. shared promising results proving that in patients with left ventricular dysfunction after acute myocardial infarction treated with trandolapril, the incidence of AF was reduced [80]. Atrial fibrillation was significantly more frequent in the placebo group than in the trandolapril group (5.3%, n = 42, compared to 2.8%, n = 22) [80]. On the other hand, however, in a small (n = 98) Japanese study, irbesartan had no advantage over amlodipine in the AF incidence reduction in a 6-month follow-up [81].

Inhibitors of XO or NADPH-oxidase, free-radical scavengers, UATs, and NLRP3 inflammasomes may be used as a means of controlling serum UA levels and lowering the risk of hyperuricemia-induced AF [52]. Future studies with a more precise design, clear study group choice and proper follow-up period are necessary for a more profound understanding of the theme and development of potential therapeutic interventions. So far, the investigations are limited by one-time measurements, cross-sectional design, and studies of populations with many other cardiovascular diseases (CVDs). The pathomechanistic insight into AF based on metabolomic research could help to identify the substrate and enable patient selection for preventive therapy.

## 3. Role of Metabolites in Atrial Fibrillation in Pre-Clinical Models

Although AF itself is not naturally observed in animals, induced arrhythmia models have significantly advanced our understanding of AF pathophysiology. Over recent decades, metabolomic studies in animal models have enabled the identification of potential biomarkers, elucidated metabolic pathways involved in arrhythmogenesis, and informed the development of new therapeutic strategies.

One of the first studies, conducted in 1986 by Uno et al., explored the relationship between biomarkers and the risk of AF in canine plasma. They revealed that cyclic guanosine monophosphate levels in dogs with induced AF significantly increased during the first week and returned to baseline after a few weeks [83]. This highlights the role of purines in the role of AF pathogenesis and suggests that the metabolomic changes are reversible [83]. However, these findings were not consistently replicated. Later, in 2000, Ausma et al. proved that in goat atrial tissue, after an initial decline over eight weeks of induced AF, the phosphocreatine level stabilized by week sixteen, while levels of creatinine, adenosine triphosphate (ATP), diphosphate (ADP), and monophosphate, as well as guanosine di- and triphosphate and nicotinamide adenine nucleotide (NAD) did not change significantly [84].

De Souza et al. conducted the first metabolomic study in animals and discovered that ADP, ATP, taurine, alanine, betaine, glucose, glutamate, NAD + NADH (reduced form of NAD), and taurine levels rose and α-ketoisovalerate levels fell in canine atrial tissue with HF following two weeks of AF [85]. This demonstrated the role of metabolic stress, inefficient energy utilization, and a shift from glycolysis to alpha-ketoacid metabolism in arrhythmogenic atrial remodelling and cardiomyopathy.

Animal models also facilitate research on AF under strictly controlled conditions such as examining the effects of various dietary patterns, sleep disturbances, or drug-induced changes on arrhythmic course, therefore recognizing future targets of therapy. A recent study by Zuo et al. found that sleep-deprived mice exhibited reduced levels of butanoate metabolism intermediates (i.e., short-chain fatty acids) in plasma, alongside significant alterations in gut microbiota composition [86]. These findings suggest that the interaction between gut microbiota and atrial metabolism may be a target for mitigating sleep deprivation-induced AF vulnerability. Fang et al. demonstrated, in a faecal microbiota transplantation (FMT) mouse model, that decreased linoleic acid levels (LA) were associated with FMT-induced AF, confirming the causal role of dysbiotic gut microbiota in AF [87].

A French study investigating the dietary impact on atrial metabolism, the metabolic profile, and the phenotype of atrial myocardium in mice showed that during a prolonged high-fat diet, a predominance of long-chain lipid accumulation and beta-oxidation activation in obese mice was present, therefore highlighting the vulnerability to AF by energy metabolism transformation [88].

In a sheep model of persistent AF, glucose was upregulated while lipid metabolism was downregulated [89]. Major changes included an increase in plasma levels of 2-phosphoglyceric acid, 1,3-bisphosphoglyceric acid, pyruvate, uridine, or citrate, alongside decreased levels of phosphatidic acid, phosphatidylcholine, phosphatidylglycerol, glycerophosphoglycerophosphates, glycerophosphoinositols, and glycerophosphoserines [89]. Interestingly, eplerenone (mineralocorticoid receptor antagonist—MRA, a type of potassium-sparing diuretic) partially reversed the metabolic shifts, increasing levels of acylcarnitines and lysophospholipids, phospholipids, and neutral lipids [89]. These findings suggest that eplerenone may have the potential for AF prevention and supportive treatment.

Regarding AF recurrence after ablation, Yoshio et al. used a sheep model to demonstrate that Galectin (Gal)-3 serum levels are higher in patients with persistent AF compared to paroxysmal AF, and Gal-3 predicts atrial tachyarrhythmia recurrences following a single ablation [90]. Targeting transforming growth factor beta–mediated signalling to reduce myofibroblast activation and atrial fibrosis may lower the total AF burden, making this pathway a promising therapeutic target [90].

While animal models offer valuable insights into AF mechanisms and potential therapies, the multifactorial and progressive nature of AF limits the generalizability of these findings. Therefore, data extrapolation should be approached with caution, despite promising results. Notably, the multifactorial, slow-progressing character of AF restricts the generalizability of animal models; therefore, extrapolating data should be done cautiously, even while animal study results appear promising.

## 4. Metabolomics-Based Findings in Clinical Studies

A growing body of evidence discusses metabolite alterations using metabolomic analyses for AF prediction. Several authors established specific metabolic patterns in AF [47,91,92,93,94,95,96,97,98,99,100,101,102,103,104,105,106,107,108,109,110,111,112,113,114,115,116,117]. Table 1 presents the current state of knowledge on the metabolomics investigations in AF. The table is ordered by time of publication—from the oldest to the most recent studies. The detailed information is related to the studied population, biological material, key results, and possible clinical implications. The majority of studies investigated plasma samples due to their clinical availability with less invasive harvesting procedures. However, some authors focused on tissue analyses—left atrial appendages allowing for a better pathophysiological understanding of AF mechanisms.

The search was performed in the PubMed database, including studies from 2008 to 2024. The keywords were “atrial fibrillation” combined with “metabolomics”.

Proper myocardial function highly depends on sufficient oxygen supply. Cardiac metabolism involves the oxidation of fatty acids (FAs) as the main source of energy, followed by glucose oxidation and glycolysis [118]. The metabolic state is altered in AF, so notable changes in oxygen delivery and energetic sources are observed [119]. In subsequent paragraphs, metabolomic analyses in AF in clinical studies are discussed, considering particular groups of compounds and including variations in the metabolism of glucose, amino acids, and lipids as well as their derivatives, purine, among others. Novel biomarkers for AF prediction and novel aspects of the pathophysiological background of AF are proposed. Also, potential therapeutic interventions such as modern precision medicine are highlighted and commented on. However, in one study by Ko et al., investigating plasma samples of 2458 European ancestry participants from the Framingham Heart Study through liquid chromatography with tandem mass spectrometry, none of the metabolites tested were significantly associated with the risk of future AF [93]. In their metabolomic profiling, glycocholate, glycodeoxycholate, and bile salts were identified but the compounds did not substantially correlate with the risk of NOAF [93]. Nevertheless, the authors recognize that the study faces several important limitations. First, metabolites outside the targeted platform might have been missed and the results might have been skewed toward the null due to a significant misclassification caused by the coefficient of variation of several of the molecules. Moreover, subtle correlations may have been obscured by the rigorous threshold for adjusted p values and the study might have had limited ability to identify tiny effect sizes. Also, there was significant diversity in the study designs, material, and studied population which might have resulted in the under-recognition of some tissue-specific metabolite profiles. Finally, regarding the fact that AF often goes undetected in clinical settings, the incidence of newly diagnosed AF may have been underreported in this study and due to different subtypes of the arrhythmia (paroxysmal, permanent, and persistent), specific metabolite associations might have been missed [93].

### 4.1. Lipid Metabolism in Atrial Fibrillation

Lipids are essential in the human body as mediators in various signalling pathways, components of membranes, and important energy sources [120]. Lipid metabolism is complex and includes the metabolism of fats, waxes, sterols, fat-soluble vitamins, glycerides, phospholipids, and other compounds. Fatty acids are carboxylic acids with a variable number of carbon atoms (from two to over thirty) connected with an aliphatic chain [121]. The simplest clinical classification is based on the saturation of the chain, meaning the presence of single bonds between the carbon atoms (C=C). Herein, saturated fatty acids (SFAs) with no double bonds, and the group of unsaturated FAs, monounsaturated fatty acids (MUFAs) and polyunsaturated fatty acids (PUFAs), possessing one or more double bonds, respectively, can be distinguished [121].

The source of SFAs is the consumption of animal fats and plant oils in the diet; however, the human body can synthesize them on its own as well, in a process called lipogenesis, when carbohydrate excess is present [122]. Unsaturated FA concentration, on the other hand, depends only on external supplementation.

Circulating fatty acids (FAs), under physiological conditions, after entering cardiomyocytes, undergo oxidation to produce nicotinamide adenine dinucleotide, flavin adenine dinucleotide, and also, like with glucose oxidation, acetyl coenzyme A (acetyl-CoA) [123]. Both pathways are independent of the production of the substrate. Of note, increased FA oxidation suppresses glucose metabolic changes and vice versa [123]. Acetyl-CoA is the primary substrate for the tricarboxylic acid (TCA) cycle (Krebs cycle)—the major energetic source of the cardiomyocyte through oxidative processes for adenosine triphosphate (ATP) production.

Although SFAs are essential for human life [123,124], on the other hand, they have also been identified as a critical risk factor for many CVDs, including AF [125]. Excessive SFA or carbohydrate consumption has been linked to altered lipid metabolism and elevated low-density lipoprotein-cholesterol concentrations [126]. Furthermore, it has been shown that SFAs, especially palmitic acid, may induce oxidative stress, mitochondrial dysfunction, and adipose tissue inflammation, an effect called lipotoxicity [127,128]. Therefore, with its elevated level, an increased risk of AF is observed [125,129]. Unlike SFAs, MUFAs, indispensable ingredients of the Mediterranean diet, reduce the risk of CVD and AF [129]. With PUFAs, which are integral parts of cellular membranes and modulate their fluidity as well as taking part in signalling processes, literature findings about their causal role in AF are inconsistent [130].

During AF, there is an increased catecholaminergic response [131], which stimulates lipolysis, raising the amount of free FA in circulation and their uptake by cardiomyocytes, finally leading to oxidative stress via increased ROS production under hypoxia [132]. Among the lipid-derived inflammatory mediators are oxylipids, phospholipids, and neutral lipids [132]. The robust inflammatory reaction involving adipose tissue includes the activation of phospholipase A2 and the release of arachidonic acid and its derivatives—eicosanoids (i.e., prostaglandins) and endocannabinoids [133]. Arachidonic acid is metabolized by cyclooxygenase, lipooxygenase, and cytochrome P450 pathways further forming prostaglandins, hydroxyeicosatetaneoic acids, thromboxane, lipoxins, and epoxyeicosatrienoic acids (EETs). EETs by themselves were shown to have cardioprotective effects, acting as anti-inflammatory and pro-fibrinolytic agents [134]. However, their further metabolization to dihydroxyeicosatrienoic acids by soluble epoxide hydrolase (sEH) leads to atrial remodelling [134]. The exact molecular mechanisms remain unexplored; however, it could be assumed, that therapy targeting the synthesis of eicosanoids could limit AF burden by interrupting the lipolytic capacity and the adipose tissue toxicity [135]. Recent studies demonstrated that treatment with sEH inhibitors decreases arrhythmia inducibility in pre-clinical in vivo models [135], therefore suggesting future research designs with the use of metabolomics. Increased levels of EETs may decrease atrial structural and electrical remodelling, electrical instability, and pro-arrhythmic substrate, therefore contributing to AF prevention [135].

Available data recognize that changes in lipid concentrations can influence cellular function and provoke inflammatory responses [132,136]. In patients with myocardial infarction (MI), a strong positive correlation between lipid metabolites and inflammation was found [136]. Similarly, in AF, the differences in fatty acids and phospholipids levels in persistent AF patients compared to healthy controls indicate enhanced inflammation and the role of oxidative stress in the pathogenesis of the disease [95]. In a study by Jung et al., all FFAs were downregulated in AF patients [95]. Changes in fatty acid composition within specific lipid classes, including FFAs and phospholipids, were influenced by their degree of unsaturation. The levels of PUFAs and MUFAs were lower in AF patients, contrary to SFAs [95]. Moreover, the patterns were similar in recurrent and non-recurrent AF [95]. These findings could be the background for future research on the supplementation of unsaturated FAs for AF prevention in some groups of patients based on metabolomic profiles.

Another study reported that faecal samples of AF patients revealed a reduction in faecal short-chain FA (acetic, butyric, and propionic acids) levels, with a declining trend from paroxysmal to persistent AF [109]. The recent metabolomic analysis confirmed the enrichment pathways of unsaturated FA biosynthesis, revealing the downregulation of arachidonic and palmitelaidic acid, making them potential biomarkers for paroxysmal AF [116]. In another study by Zhou et al., 16 lipids were dysregulated in AF patients and had a possible impact on arrhythmia onset [98]. Among them, FA 20:2 and FA 22:4 showed a strong correlation with the size of the left atrium and, therefore, could be perceived as a prognostic marker for early-stage AF monitoring and/or arrhythmia prognostic evaluation [98]. In a general population cohort of 2059 adults, glycerol, cholesterol ester 16:1, and phosphatidylcholine 32:1 mediated the association of CVH score with incident AF [114]. On the other hand, a recent case–control study using an ultra-high-performance/pressure liquid chromatography system coupled to a mass spectrometer did not associate a single lipid with AF incidence [115]. In a study reported by Lu et al. [111], higher levels of sphingomyelin d18:1/14:0 in the control group compared to AF patients were found, which promotes the abovementioned theory.

Recently, Emmert et al. found a lower concentration of lysophosphatidylcholine (LPC) a C 20:3 in AF cases [107], which is in line with the available data [95,111], making it a possible biomarker for AF prediction.

Existing evidence suggests that LPC has a role in inflammatory responses and the progression of metabolic diseases [107,137]. Decreased levels of LPC species have been shown to contribute to protective effects in both anti-inflammatory responses and the advancement of metabolic disorders. LPC can stimulate the migration of lymphocytes and macrophages, boost the production of pro-inflammatory cytokines, trigger oxidative stress, and promote apoptosis, all of which can exacerbate inflammation and contribute to disease progression [137,138]. Its impact on endothelial cells, vascular smooth muscle cells, and arteries is crucial in the development of atherosclerosis and other cardiovascular conditions [137,138]. As a result, targeting LPC and lipid metabolism could offer a promising therapeutic approach for diseases related to inflammation [138]. In a high throughput lipidomics-based liquid chromatography–mass spectrometry analysis in AF patients, lysophosphatidylcholine 20:5, fatty acid 23:0, and phosphatidylinositol 16:0_18:1 were defined as a combinational biomarker to distinguish AF patients from the controls [108]. All of these findings are highly suggestive of the potential role of lipid metabolism alterations in AF occurrence.

Some authors suggest that the mechanism of valvular and non-valvular AF may differ, as the valvular arrhythmic episodes could be related to the glycolysis/gluconeogenesis pathway, and non-valvular AF to the TCA cycle [105]. Post-operative AF (POAF) seems to be a distant clinical entity with different underlying pathomechanisms as well [58,102,113]. The study conducted by Li et al. is one of the first to investigate the mechanisms of POAF using multi-omics approaches and to validate the findings in a new patient cohort [102]. The authors identify pre-existing differences in proteins and metabolites in the plasma of patients who are predisposed to POAF following coronary artery bypass grafting (CABG) [102]. The study suggests that the dysregulation of peroxisome proliferator-activated receptor alpha (PPARα) and glutathione metabolism pathways, which are associated with metabolic and redox imbalances leading to electrical remodelling, may play a central role in POAF development [102]. Additionally, lower levels of plasma phospholipid transfer protein and apolipoprotein-C3, along with higher levels of cholesteryl ester transfer protein and glutathione peroxidase 3, are associated with POAF [102]. In AF related to mitral valve disease, fatty acids and linoleic acid metabolism were interrupted [104]. These proteins and metabolites could potentially serve as biomarkers for predicting POAF [104]. In the group of patients after coronary artery bypass grafting (CABG) who developed POAF, a lower ratio of glycolytic end products to end products of lipid metabolism was found, suggesting its role in arrhythmia pathogenesis, especially in the preoperative period [91]. The ratio, moreover, correlated positively to the time of onset of POAF [91].

From a clinical point of view, lipid disorders can be classified as dyslipidaemia, meaning an abnormal plasma level (high or low) of any or all lipids, hyperlipidaemia, which encompasses only the spectrum of increased lipid levels in the blood, or hypercholesterolemia, narrowing the group to elevated blood cholesterol. The role of broadly understood lipid disturbances in AF onset is unquestionable; however, the mechanisms are complex and not fully elucidated. Several studies suggest the paradoxical inverse correlation of total cholesterol (TC), low-density lipoprotein-cholesterol (LDL-C), and high-density lipoprotein-cholesterol (HDL-C) levels with the risk of future AF [139,140,141]. Even though dyslipidaemia increases the risk of atherosclerosis and coronary artery disease, which are recognized AF risk factors, high levels of LDL-C, TC, and HDL-C are associated with a lower risk of AF [139,140]. In a population-based study of >65,000 adults aged 45 to 60 years without any history of cardiovascular disease, using data from the Swedish National Patient Register and Cause of Death Register—the Apolipoprotein-related MOrtality RISk (AMORIS) cohort, the authors proved that higher levels of TC and LDL-C were associated with a lower risk of AF and, on the contrary, patients with lower levels of HDL-C, high triglyceride (TG) levels, and high TG/HDL-C had a higher risk of AF over 30 years of follow-up [140]. The pathomechanism could be connected to the stabilizing effect of cholesterol on cellular membranes [139]. Moreover, linking ageing with the inverse association of TC and LDL-C and the higher prevalence of AF might explain the inverse association of these lipid compounds with AF occurrence [141]. Nevertheless, hyperlipidaemia remains a significant independent CVD risk factor accounting for atherosclerosis and vascular ageing [142]. New insights are necessary for a detailed understanding of the pathophysiology behind this paradoxical observation.

To summarize, an altered lipid profile in AF was proved to increase the risk of arrhythmia development and progression [95,98,102,107,109,111,115,116]. FAs play an important role in human health and take part in the development of atherosclerosis and overall cardiovascular risk including AF [123,124,125,126,127,128,129]. Elevated SFAs are considered especially important risk factors for arrhythmia onset [125,129]. Increased FA oxidation decreases glucose oxidation, which increases the generation of lactate and protons, and causes cellular acidosis, inflammation, and oxidative stress. Therefore, lowering the FA/glucose metabolism ratio may reduce oxygen waste and increase myocardial efficiency [143]. With diet modification, which reduces SFAs and carbohydrate intake, the AF burden can be decreased [88,126,129]. Despite literature findings supporting the beneficial role of unsaturated FAs in AF prevention [130], no routine supplementation of MUFAs or PUFAs has been approved for AF management so far. However, FA composition differs between individuals and depends on proteins and gene expressions, which are influenced by dietary habits [144]. Properly designed population-based prospective trials should be designed for their real assessment to enable the clinical use of FAs in certain patients with a targeted, metabolomic approach.

With a view to the future, a new therapeutic approach, based on metabolomic research and metabolically active drugs, can be developed. Ranolazine, primarily considered an antianginal drug, improves redox balance and mitochondrial activity by suppressing FA oxidation and increasing glucose oxidation [124,145]. Therefore, with its use, supraventricular arrhythmia occurrence in patients with MI was suppressed [146]. A later trial demonstrated that the combination of a moderate dose of ranolazine and a lower dose of dronedarone was more effective than individual drug therapy at reducing the incidence of AF in the enrolled population (HARMONY TRIAL) [147], which seems promising for further AF management. Similarly, trimetazidine, another antianginal agent, decreases FA metabolism and increases glucose uptake, which provides a protective effect in myocardial ischemia [148] and may be useful in the treatment of AF [70,149]. However, the findings need to be confirmed in further research concerning metabolomic profiles to find optimal responders for the therapy.

### 4.2. Acylcarnitines in Atrial Fibrillation

Also, acylcarnitines, derivatives of FA oxidation, were found to be associated with a higher risk of incident AF [101]. Their changed level allows us to assume the poor metabolic status of cardiomyocytes, as their accumulation destabilizes cellular membranes and provokes myocardial remodelling [150], which probably has a pro-arrhythmic effect [150,151]. In a recent study by Lind, based on nontargeted metabolomics analysis (ultra-performance liquid chromatography coupled to time of flight mass spectrometry), 9-decenoylcarnitine was found to be an AF biomarker. Moreover, the compound correlated with the left atrial size and LV mass [103]. However, Mendelian randomization did not confirm its causal role in AF [103]. Krause et al. found that human-derived engineered heart tissue exposed to long-chain acyl-carnitine C18:1AC was characterized by an impairment of contractile force on the grounds of inhibiting mitochondrial respiration [152]. Further on, high serum concentrations of C18:1AC were linked to both the incidence and prevalence of AF in a patient cohort [152]. A Swedish study by Smith et al., measuring baseline fasting metabolites in 3770 AF-free Malmö Diet and Cancer Study participants, revealed that in a 23-year follow-up, 650 AF cases were identified, where seven medium- and long-chain acylcarnitines were associated with disease onset [101]. In the MURDOCK Cardiovascular Disease Study population, from the part without AF history (n = 1892), a total of 233 patients developed NOAF during follow-up and their plasma samples collected revealed higher levels of medium chain acylcarnitines, short-chain dicarboxylacylcarnitines, and long-chain acylcarnitines [97] Such findings might suggest the potential role of acylcarnitines in AF pathogenesis. A better mechanistic understanding of the onset of AF and new prospects for prevention and treatment could result from the validation of these findings in larger patient populations.

### 4.3. Amino Acid Alterations in Atrial Fibrillation

Cardiovascular physiology and pathology are fundamentally influenced by amino acids (AAs). In particular, L-arginine, L-glutamine, L-tryptophan, and L-cysteine were reported to have a significant function in vascular health through various metabolic processes [153]. Additionally to the regulation of cellular homeostasis, they exert anti-inflammatory and antioxidant effects [153]. Furthermore, AAs exert one of the primary metabolic substrates for the myocardium [154]. Emerging data indicate the alteration of AA composition is involved in AF pathogenesis and progression [47,91,94,96,110,111,113,117]; however, the exact mechanism remains unclear. Mayr et al. reported that the left atrial appendage of patients with persistent AF had higher levels of ketogenic AAs and glycine [91], indicating the energetic switch from FA oxidation as an adaptation to metabolic stress. However, the findings were not confirmed in a study by Huang et al. [117], where plasma levels of glycine were decreased. The authors investigated the correlation between AAs and age-related AF risk [117]. Additionally, decreased glycine levels and higher plasma levels of lysine, tyrosine, glutamic acid, methionine, and isoleucine were found in the group of persistent AF patients compared to controls [117]. In a further animal model, distinct AA metabolic patterns were found in ageing mice [117]. The observed AAs profile disturbances give novel insight into disease pathogenesis and progression and may suggest that AAs may participate in the development of age-related AF [117].

A total of 85 consecutive patients hospitalized for AF with a restoration of sinus rhythm at 6 months had higher plasma levels of kynurenine and kynurenine/tryptophan, while arginine and methionine/methionine sulfoxide levels were lower [47]. This may indicate potential inflammatory and oxidative stress mechanisms contributing to AF onset and the abovementioned AAs may be potential AF biomarkers [47].

D-glutamic acid, belonging to the group of D-AAs, has been reported to regulate neuronal transmission [155], and elevated levels of D-glutamic acid were found in the plasma and atrial appendage tissues of patients with AF compared to the SR control group [94]. The findings were confirmed in a previously mentioned study by Huang et al. [117] and a cohort study by Lu et al. [111], where altered metabolic pathways during AF progression included D-glutamine and D-glutamate metabolism. These findings may point to some influence of autonomic neuropathy in arrhythmia pathogenesis [94,155] and point to novel diagnostic markers for arrhythmia prediction.

In an untargeted gas chromatography–mass spectrometry metabolic analysis of AF patients, alterations of plasma AAs were found, which exhibited prediction values for arrhythmia [96]. The strongest association was found between arrhythmia and threonine [96]. With the persistence of AF, the level of circulating 4-hydroxypyrrolidine-2-carboxylic acid, a compound biosynthetically derived from proline, was progressively reduced without alterations in proline levels [96]. Proline is involved in collagen synthesis, therefore, the authors speculated that in AF, due to a defect in proline metabolism, increased collagen expression is observed, which explains the pronounced atrial fibrosis in the arrhythmia [96].

Arginine and its derivatives participate in cardiovascular oxidative and inflammatory processes [156]. In a recent study based on the liquid chromatography–mass spectrometry of plasma samples, an inverse correlation with incident AF was observed for baseline arginine, whereas a positive one was observed for N1-acetylspermidine [110]. Previous research has already reported decreased arginine levels in AF, suggesting its involvement in AF pathogenesis [157,158]. Moreover, MedDiet was proven to influence HF risk in the same study [110], which could also be investigated in the AF group. Identifying further alterations of AA homeostasis could lead to dietary interventions as a therapeutic strategy for AF.

In the group of patients after CABG, targeted metabolomic analyses of pericardial fluid and serum samples revealed that elevated levels of aceglutamide, ornithine, methionine, and arginine were significantly associated with POAF [113], contrary to findings in non-cardiac surgery patients, where decreased arginine levels were connected with AF onset [110]. The results suggest different mechanisms of POAF and such perturbations of circulating levels of AAs may be a potential target for the restoration of CV homeostasis in future AF prediction and management in this group of patients.

As can be concluded, AA alterations in AF seem to have a complex mechanism, including oxidative stress, inflammation, and autonomic neuropathy, as well as an ageing-related aspect. POAF seems to be a distinct clinical entity. Further investigation confirming current findings could be of particular interest for the management of arrhythmia, as finding novel biomarkers may help in disease prediction and the implementation of proper therapy.

### 4.4. Ketone Body Metabolism in Atrial Fibrillation

Ketone bodies (acetoacetate, β-hydroxybutyrate, and acetone) are organic compounds used as alternative energy substrates for ATP production, which are produced in the liver from FAs. They are utilized by tissues by the way of transformation to acetyl-CoA, which enters the TCA cycle [159]. In the very first metabolomic study, by Mayr et al., in patients with persistent AF undergoing cardiac surgery, a rise in β-hydroxybutyrate along with ketogenic amino acids (notably tyrosine and leucine) was found in atrial appendage samples [91]. The findings were confirmed by Brown et al. [160], which may suggest some degree of the organism’s adaptation to the arrhythmia. In animal models of ventricular-tachypaced dogs who developed AF-induced heart failure (HF), increased metabolic stress was observed along with the shift from glycolysis to keto acid metabolism [85]. The oxidation of ketones as the only energetic compound was proven to correlate with impaired cardiac contractility [161] and pro-arrhythmic effects due to cardiac fibrosis and consequently hypertrophy and inflammation [162]. Therefore, with these metabolomic findings, the self-perpetuating nature of AF may be explained, suggesting the potential role of ketone bodies in atrial cardiomyopathy.

### 4.5. Purine Metabolism in Atrial Fibrillation

The purine metabolic pathway is strongly implicated in AF onset and progression in complex pathomechanisms [47,98,112]. Among others, uric acid, the end product of purine catabolism, already broadly commented on in this article, and adenosine, fundamental nucleoside building blocks of nucleic acids, have been reported to correlate with AF incidence [59,61]. In a recent pilot study by Soulat-Dufour, prospectively evaluating patients hospitalised for AF with the restoration of SR at 6 months, higher levels of urea/creatinine, along with other inflammatory markers, were found in patients who developed AF and correlated with the decreased median left atrial reservoir strain, also indicating atrial cardiomyopathy and the worsening of cardiac function [47]. LC–MS-based untargeted metabolomics with a targeted quantification assay by Zhou et al. revealed 24 dysregulated metabolites in untreated AF patients, where eight of them were found to belong to the purine metabolic pathway [98]. In the study, uridine 5′-diphosphate, adenine monophosphate (AMP), uridine 5′-monophosphate, hypoxanthine, para-aminobenzoic acid, adenine, adenosine 3′-monophosphate, and guanosine 5′-monophosphate displayed positive correlation expression patterns [98]. Furthermore, in a bioinformatic analysis, seven metabolites (adenine, 3′-AMP, adenosine 3′ 5′-diphosphate, adenosine monophosphate, guanosine monophosphate, hypoxanthine, and inosine) were upregulated in AF patients [98]. In patients with persistent AF undergoing a surgical ablation of the arrhythmia, alterations of adenine in the left atrial appendage were observed [112]. Such findings suggest that the purine pathway might play a role in the progression of AF disease and that these metabolites might be used to create an analytical panel of biomarkers to diagnose AF. Moreover, agents decreasing serum uric acid levels, i.e., allopurinol, could be of particular clinical interest in terms of AF prevention. However, so far, the results of conducted studies are inconclusive and do not support its routine use [163].

### 4.6. Other Metabolomic Findings in Atrial Fibrillation

In AF, metabolomics may serve to discover new disease-risk biomarkers and alterations in the metabolic pathways [47,91,92,93,94,95,96,98,99,100,101,102,103,104,105,106,107,108,109,110,111,112,113,114,115,116,117]. Elevated secondary bile acid concentrations of glycolithocholate sulphate and glycocholenate sulphate, independently of other CVD risk factors, including liver diseases, were a predisposing factor for AF development in blacks in the Atherosclerosis Risk in Communities (ARIC) cohort [92]. In a study reported by the same authors, the findings were partially replicated in white and black ARIC communities [99], indicating some origin-based mechanisms. Glycocholenate sulphate was associated with AF in the replication and combined samples, but glycolithocolate sulphate was not related to AF risk in the replication sample [99]. Moreover, pseudouridine, uridine, and acisoga, a Polyamine metabolite, were associated with AF [99].

In atrial appendage and plasma samples analysed in liquid chromatography positive ion electrospray ionization tandem mass spectrometry, patients with and without AF were found to have different metabolic profiles reflected in 24 serum metabolites [94]. In this study, in atrial appendage and plasma samples, creatinine, D-glutamic acid, choline, hypoxanthine, and niacinamide were thought to be significant characteristics of AF patients [94]. In a Chinese study using the multi-omics technique, precise metabolic, phenomic and genomic data on potassium and sodium ions, chitin, benzo[a]pyrene-7,8-dihydrodiol-9,10-oxide, and Celebrex (TN) were associated with AF risk [100]. Plasma samples of 3770 participants in the Malmö Diet and Cancer Study pointed to caffeine and acisoga as risk factors for AF occurrence, whereas beta carotene was associated with a lower risk [101]. In a Spanish study, investigating the associations of kynurenine-related metabolites with the risk of HF and AF, quinolinic acid was correlated with increased AF risk [106]. The MedDiet intervention had an impact on the metabolic associations in AF [106]. The higher levels of kynurenine and kynurenine/tryptophan were also found in further studies [47]. Differences between the countries may indicate some population and environmental-specific mechanisms. In the left atrial appendage of persistent AF patients undergoing surgical ablation, multiple pathways related to mitochondrial energy metabolism were enriched, including alterations of raffinose, adenine, and D-mannitol [112]. Hu found different metabolites related to valvular AF compared to non-valvular AF [105]. The valvular one was especially related to 20-hydroxy-PGF2a and glutathione, part of the glycolysis/gluconeogenesis pathway, whereas non-valvular AF was related to thymine and thiopental, contributing to the TCA cycle [105]. The findings may suggest that these two subgroups of AF constitute different clinical entities with different pathomechanisms, similar to POAF [58,102].

Metabolomic analyses allow for more profound insight into AF pathophysiology, allowing for the discovery of novel biomarkers for disease prediction. However, most of the research conducted so far has a cross-sectional character and provides untargeted analyses. Not many of the results were replicated. The subject needs to be further investigated in order to improve patient management and decrease mortality.

## 5. Clinical Scenarios in Atrial Fibrillation Metabolomics: Translating Metabolomic Findings into Diagnostic and Therapeutic Workflows

Metabolomic assessment has emerged as a diagnostic and prognostic tool in various cardiovascular diseases, frequently leading to possible future therapies [21,28,30,116]. Pathophysiological links between AF and its consequences such as HF, through disturbed metabolic pathways, have been proposed [85,164,165,166,167,168]. Metabolomics provides the potential to bridge research findings with clinical applications. Specifically, the following clinical scenarios are linked to AF and highlight its translation into routine workflows.

### 5.1. Atrial Fibrillation-Induced Heart Failure: Metabolomic Considerations

HF is considered the most severe adverse event caused by AF [164,169] with a cumulative incidence of 20% at 5 years [165]. Moreover, the combination of AF with HF is associated with a deterioration in quality of life and prognosis, and a higher risk of death [165,166,167]. AF and HF coexist in clinical practice and exacerbate each other [166,167]. The potential link between AF-induced HF was linked to atrial remodelling, leading to cardiomyopathy and contractile dysfunction [168]. Some authors suggest the role of oxidative stress in HF [170,171], which also links it to AF mechanisms [33,44,46,49]. Decreased nicotinamide adenine dinucleotide (NAD^+^) levels, an important molecule for ATP production and ROS detoxification, were found in both AF and HF patients and correlated with mitochondrial and cardiomyocyte dysfunction [172,173,174]. Notably, the supplementation of NAD^+^ or its precursor nicotinamide ribosome (NR) has emerged as a novel metabolic therapy in HF patients [175,176,177]. In experimental studies, NR supplementation was proven to prevent contractile dysfunction [172,173,178,179,180]. However, the results need to be confirmed in clinical trials.

Other hypotheses linking AF with HF included inflammation [106,110,181,182]. In a population at a high risk of CVD, the disturbances in the tryptophan–kynurenine pathway, related to inflammatory response, were associated with both AF and HF [106]. Increased kynurenic acid levels, along with homocysteine, were found in patients with persistent AF and correlated with aortic stiffness [181], indicating a pro-inflammatory response. Arginine derivatives, connected with oxidative and inflammatory processes, are also involved in AF and HF pathophysiology [110]. Moreover, through metabolic mediations, the Mediterranean diet was proven to lower the incidence of AF and HF [106,110].

Metabolomic analyses of cardiac tissue from ventricular-tachypaced congestive HF dogs revealed increased concentrations of glucose and alanine [85]. A combination of metabolic stress, inefficient energy use, and a switch from glycolysis to alpha-ketoacid metabolism was suggested by the accumulation of ADP/ATP and the depletion of alpha-ketoisovalerate at the 2-week mark [85]. Further clinical studies of plasma samples using the liquid chromatography–mass spectrometry method showed that in patients with AF-induced HF, glycolysis or gluconeogenesis, tyrosine metabolism, and the pentose phosphate pathway were enriched [183]. Zhang et al. found 10 differentially expressed metabolites, which could be potential biomarkers in HF patients compared to AF without HF [183]. Recently, acisoga, the polyamine metabolite originating from arginine, was proven to be a novel biomarker for decreased LVEF [184]. Its increased levels correlated with HFrEF, revealing a strongly negative correlation with left ventricular strain and therefore systolic function [184]. In the Atherosclerosis Risk in Communities [ARIC] study, its elevated levels correlated with the onset of AF [92]; hence, the metabolite could be the potential link between these two entities. Therefore, metabolomic analyses seem promising in HF patients with AF in predicting arrhythmia and targeting energy metabolism by dietary interventions. Notably, the atrial cardiomyopathy is reversible, as after reversal to sinus rhythm, cardiac remodelling withdraws [185]. Therefore, proper AF management and the prevention of its consequences is of high importance. The quick prediction of arrhythmia would decrease the rate of AF-induced HF and decrease morbidity. Further studies are necessary to validate and complement the current findings.

### 5.2. Metabolomic Alterations Associated with Post-Myocardial Infarction Atrial Fibrillation

Atrial fibrillation is the most frequent arrhythmia following acute myocardial infarction (AMI), occurring in 6–21% of these patients [186]. The presence of AF significantly worsens the clinical outcome, increasing mortality and reducing quality of life, regardless of arrhythmia type [186,187,188,189], making it a critical clinical issue. In a study by Raczkowska-Golanko et al., new-onset AF (NOAF) patients exhibited the highest rate of ST-elevated AMI (40%), the longest hospitalization, and the highest in-hospital mortality rates [188].

The peri-infarct period is associated with an increased risk of AF; however, the exact pathophysiology and clinical significance remain debated and are not yet fully understood. AMI induces acute physiological stress, triggering a generalized metabolic reaction. Tissue hypoxia leads to a decrease in cardiac output, hypoxemia, and metabolic acidosis. AF typically results from post-AMI atrial ischemia, which leads to oxidative stress and inflammation, and consequently, elevated filling pressures, volume overload, and atrial stretch [186,189]. Inflammation further stimulates atrial fibrosis formation and dilatation, leading to atrial cardiomyopathy and a substrate vulnerable to AF creation and perpetuation [20,70].

Despite these associations, no studies have specifically identified a biomarker for post-MI AF, highlighting a key area for future research. Given the poor prognosis in this patient group and the therapeutic challenges of balancing ischemic and haemorrhagic risks, identifying metabolomic biomarkers for AF prevention in post-MI patients could have significant clinical implications. Importantly, the distinct clinical entities of ST-elevation MI (STEMI) and non-ST elevation MI (NSTEMI) should be considered separately, as their underlying mechanisms differ.

Nevertheless, numerous studies have reported metabolomic changes in MI [190,191,192,193,194,195,196,197,198,199]. Various compounds have been proposed as potential biomarkers for both, MI and AF. For example, increased LPCs, which are linked to atherosclerosis pathophysiology [190] (a risk factor for AF), have been associated with MI prediction [191]. Zhang et al. suggested a role of eicosanoids in cardiac injury and repair after ST-elevation MI (STEMI), observing elevated plasma levels of prostaglandin E2, prostaglandin D2, thromboxane 2A, epoxyeicosatrienoic acids, 20-hydroxyeicosatetraenoic acid, and 20-oxo-leukotriene B4 [192]. Markin et al. identified significant changes in tryptophan metabolism in STEMI patients, including increased levels of anthranilic acid and tryptophol and decreased levels of xanthurenic acid and 3-OH-kynurenine [193]. Another study of STEMI patients identified 10 metabolites as potential early, sensitive, and specific disease biomarkers, including L-aspartic acid, L-acetylcarnitine, acetylglycine, decanoylcarnitine, hydroxyphenyllactic acid, ferulic acid, itaconic acid, lauroylcarnitine, myristoylcarnitine, and cis-4-hydroxy-D-proline [194]. In the same study, specific biomarkers for NSTEMI included L-aspartic acid, arachidonic acid, palmitoleic acid, D-aspartic acid, and palmitelaidic acid [194]. Other studies, without specifying MI type, identified increased levels of L-homocysteine, N-methyl arachidonic amide, sulfinic acid, cysteic acid, arginine, tryptophan, taurine, methionine, leucine, isoleucine, valine, ornithine, and 2-ketoglutarate, 5-hydroxytryptamine, acylalkyl-phosphatidylcholine C36:3, or diacyl-phosphatidylcholines C38:3 and C40:4 as potential MI serum biomarkers [195,196,197,198,199]. In most studies, higher plasma levels of metabolites associated with pyrimidine, methionine, and arginine metabolism were observed in MI patient samples.

Future prospective and well-designed metabolomic research is necessary to better understand the pathophysiology of post-MI AF, enable early risk stratification, and improve arrhythmia prevention strategies.

## 6. Is Atrial Fibrillation Linked to Gut Microbiota Dysbiosis?

Increasing evidence highlights the significant role of gut microbiota (GM) in maintaining human body homeostasis [200,201,202]. GM is the collection of microorganisms, including bacteria, archaea, and fungi, which inhabit the gastrointestinal tract. The intestinal segments with the highest microbial biomass are the proximal colon and the caecum. In healthy individuals, 90% of GM consists of *Firmicutes* and *Bacteroidetes*, and their ratio (F/B) is a relative measurement of intestinal microbial health (lower F/B ratio) or disease status (greater F/B ratio) [200]. The alterations in the F/B ratio are caused by lifestyle, i.e., diet, sleep patterns, drugs, and physical activity [202]. Some authors, however, also suggest the causative role of genetics in GM dysbiosis [203].

**Figure 2:**
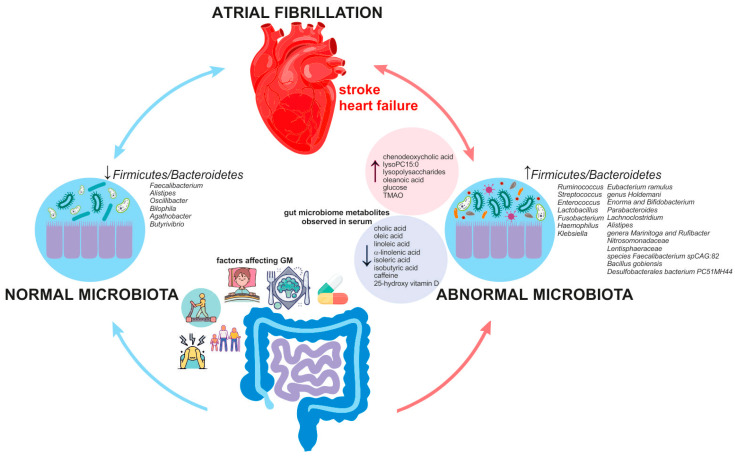
Gut microbiota dysbiosis in Atrial Fibrillation. Overgrowth of *Firmicutes* along with depletion in *Bacterioides* is observed, leading to AF onset, and/or its further consequences. On the other hand, AF episodes may trigger GM dysbiosis. GM—gut microbiota, lysoPC 15:0—lysophosphatidylcholine 15:0, TMAO—trimethylamine N-oxide, ↑ rise in, ↓ decrease in.

GM, apart from the local impact on the gut, influences various human body systems which results in the establishment of gut–lung, gut–brain and also heart–gut axis concepts [204,205,206]. Accumulating data prove the GM causative role in the pathogenesis of various CVDs, including type 2 diabetes, hypertension, and atherosclerosis [206,207,208]. Furthermore, GM dysbiosis-induced CVD may cause atrial remodelling, and, subsequently, trigger AF occurrence [209,210]. Notably, patients with AF often have the same modifiable risk factors as ones for coronary artery disease, obesity, hypertension, and diabetes, all of which are substantially linked to dietary habits and therefore GM composition [210]. The diet can target the AF substrate itself and inhibit risk factors that encourage the development of the AF substrate [210]. Moreover, GM is involved in producing numerous key biological compounds, including SCFA, trimethylamine, carnitine, and indoxyl sulphate [201]. Also, GM enzymes take part in BA metabolism, producing both unconjugated and secondary BAs [201]. These compounds affect AF occurrence as described previously in the current review. Data indicate that some microbial metabolites, i.e., trimethylamine N-oxide, FAs, or lipopolysaccharides correlate with AF onset and/or prognosis [211]. Therefore, AF incidents are supposed to correlate with GM dysbiosis [109,209,211,212,213]; however, its extent and precise mechanisms remain unclear. Figure 2 presents bacterial alterations involved in GM dysbiosis in AF and its consequences.

Zuo et al., in their recent series of studies based on metabolomic and metagenomic analyses, described the alterations in the structure and function of GM in AF patients [109,209,211,212,213]. In the cohort study, including 100 individuals, alterations in GM included a significant increase in microbial diversity and a particular disruption in the composition of the gut microbiota [212]. In faecal samples of AF patients, an overgrowth of *Ruminococcus*, *Streptococcus*, and *Enterococcus* was found, with a simultaneous decrease in *Faecalibacterium*, *Alistipes*, *Oscillibacter*, and *Bilophila* species [212]. Moreover, correlated metabolic pattern changes were observed, which could be used for AF identification [212]. AF-enriched compounds included a rise in chenodeoxycholic acid and lysoPC (15:0), along with a decrease in cholic acid, oleic acid, linoleic acid, and α-linolenic acid serum levels [212]. In another Chinese study by Chen et al., the bacterial community structure, the relative abundance of various taxa, the GM phylogenetic diversity, and the β-diversity were all altered in AF patients [214]. Compared to the SR group, there was a notable decrease in *Bifidobacterium* and an increase in *Lactobacillus*, *Fusobacterium*, and *Haemophilus* in the AF group [214]. Isoleric and isobutyric acids had a negative correlation with the quantity of *Haemophilus* [214]. The Mendelian randomization analysis revealed that two microbial taxa were causally associated with AF: the species *Eubacterium ramulus* and genus *Holdemania* [215]. In a recent population-based study, both prevalent and paroxysmal AF was associated with the genera *Enorma* and *Bifidobacterium* [216].

In other studies by Tabata et al., GM diversity was lower in AF patients, although the diversity of gut microbiota did not differ between the AF group and healthy controls [217]. At the genus level, *Enterobacter* was depleted, while *Parabacteroides*, *Lachnoclostridium*, *Streptococcus*, and *Alistipes* were enriched in AF patients compared to control subjects [217]. Moreover, the AF patients had a higher dietary intake of n-3 polyunsaturated fatty acids and eicosadienoic acid, contributing to AF pathogenesis [217].

Ageing is one of the most significant risk factors for AF [1,2,4]. Lately, aging-related GM dysbiosis has been suggested as a potential mechanism [218,219]. A recent study by Liu et al. found that, in comparison to young rats, older rats had a greater incidence of AF [219]. Furthermore, disrupted GM, specifically a decreased F/B ratio, and lower faecal SCFA levels were found to be lower in aged rats [219]. In the study of Zhang et al., it was demonstrated that age-related GM dysbiosis increases the risk of AF through an increase in levels of circulating glucose and lipopolysaccharides as well as the activation of atrial NLRP3 inflammasome [203]. Age-related increased intestinal permeability and fibrosis may play a key role in arrhythmogenesis, therefore making the GM composition a potential therapeutic target for AF prevention [203].

In another study by Zuo et al. [209,211], the authors investigated GM patterns in paroxysmal and persistent AF, proving that even though the general dysbiotic pattern was shared in all AF subtypes, certain bacteria were differently enriched at different AF duration times [209]. In different AF types (paroxysmal vs. persistent), a similar GM dysbiosis pattern was observed, and a similar trend of enterotype distribution, including a higher percentage of the enterotype *Bacteroides,* was discovered [211]. Furthermore, in persistent AF (psAF), no significant difference in GM diversity was found based on a short or long duration of arrhythmia; however, there was a notable difference in alpha and beta diversity between psAF patients and controls, indicating that psAF patients develop GM dysbiosis at an early stage [211]. Such findings could lead to the development of early-stage treatment approaches for atrial fibrillation that target the GM and postpone the arrhythmia progression. Additionally, LC–MS metabolomic analyses revealed that pro-inflammatory bacterial species like *Ruminococcus* and *Streptococcus* showed a favourable correlation with AF-enriched metabolites, such as chenodeoxycholic acid, that were enriched in PAF and psAF [211].

Concerning further metabolomic analyses in GM dysbiosis, in another small (48 individuals) cross-sectional study of Zuo et al., based on faecal GS-MC analysis with the subsequent creation of a varied-diet mice model (SCFA deficient vs. dietary fibre enriched), the authors proved that a diet rich in SCFA alleviates AF development through various inflammatory signalling pathways [109]. SCFAs, important energetic substrates and anti-inflammatory agents, are produced by the bacterial fermentation of nondigestible carbohydrates (so-called dietary fibre). Therefore, an increased intake of dietary fibre could alleviate or decrease AF episode severity and, on the other hand, a deficiency in SCFA production may worsen the progression of the disease [109]. Therefore, understandable scientific interest is in prebiotics and probiotics that modulate GM [220,221] and which may have beneficial effects on CV diseases and AF onset [109,200]. Various therapies can be employed to avert the harmful biological consequences of dysbiosis [217,219]. Approaches include the consumption of food products that foster the growth or activity of beneficial microorganisms (prebiotics), direct oral ingestion of healthy live microorganisms (probiotics), or application of gut microbiota-derived metabolites like SCFAs (postbiotics) [220]. Also, changes in dietary habits significantly contribute to fluctuations in gut microbiota composition [217]. High-fat and high-sugar diets cause alterations in gut permeability that let bacterial lipopolysaccharides (LPSs) move from the gut into the bloodstream, resulting in systemic endotoxemia, inflammation, and oxidative stress [217]. Therefore, a high ingestion of dietary fibre seems to benefit the GM composition. Recent investigations have shown that various drugs and gut bacteria interact in both directions and that this interaction may also affect how drugs are metabolized [217]. The medication includes non-steroidal anti-inflammatory drugs, digoxin, calcium channel blockers, and antibiotics [217,219].

Finally, the authors investigated the GM composition of AF recurrence (RAF) after the arrhythmia ablation [213,222], constructing a GM-based predictive model for RAF [26]. The GM composition and metabolomic profile of patients with recurrent AF (17 individuals) and the non-RAF group (23 individuals) were significantly different from non-AF controls [213]. Microbes associated with RAF included the genera *Marinitoga* and *Rufibacter*, the families *Nitrosomonadaceae* and *Lentisphaeraceae*, and the species *Faecalibacterium spCAG:82*, *Bacillus gobiensis*, and *Desulfobacterales bacterium PC51MH44* [213]. In another study of GM composition after AF ablation, by Huang et al., when compared to controls, species richness and diversity considerably increased in AF patients [222]. Among them, there was a large decrease in symbiotic bacteria like *Agathobacter* and *Butyrivibrio* and a considerable increase in opportunistic pathogenic bacteria including *Klebsiella*, *Haemophilus*, *Streptococcus*, and *Enterococcus* [222]. Moreover, the downregulation of caffeine, which had a negative correlation with *Klebsiella*, and ascorbic acid and oestradiol, which had a positive correlation with *Agathobacter*, was also observed in AF patients [222]. Following catheter ablation, most AF patients had an increase in intestinal symbiotic bacteria (*Lactobacillus*, *Agathobacter*, *Lachnospira*, etc.) and a decrease in harmful species (*Ruminococcus*, etc.) [222]. Furthermore, there was an increase in citrulline, which had a positive association with both *Lactobacillus* and *Ralstonia,* and a downregulation of oleanolic acid, which showed a negative correlation with the latter [222].

Similarly, the role of GM in POAF pathogenesis was proven recently [223,224]. Wang et al. proved that patients with POAF had a markedly altered GM composition, with a drop in *Escherichia-Shigella*, *Klebsiella*, *Streptococcus*, *Brevundimonas*, and *Citrobacter*, and an increase in *Lachnospira*, *Acinetobacter*, *Veillonella*, and *Aeromonas* species [223]. Additionally, POAF patients had lower plasma 25-hydroxy vitamin D levels, which were inversely linked with a high *Lachnospira* population [223]. In another study, by Nenna et al., the main GM metabolic product, trimethylamine-N-oxide (TMAO), was proven to be crucial for myocardial fibrosis and POAF onset, which occurred after cardiac surgery in 38% of patients [224]. Therefore, TMAO level elevation was stated as an independent risk factor for POAF [224], which may allow arrhythmia prediction and therefore its prevention through specific therapeutic interventions targeting GM.

Despite the available data that strongly support the theory of a relationship between GM dysbiosis and AF, direct evidence connecting gut-associated metabolites to AF susceptibility is still missing. None of the studies considered the influence of comorbidities, lifestyle, and drugs on the changes in GM. Also, the AF population had not been selected specifically. Larger randomized trials comparing groups with and without adjusted AF risk factors and intestinal dysbiosis are required. The subject is of particular clinical interest, as GM dysbiosis is a potentially targetable factor that might provide novel treatment avenues for AF prevention.

## 7. Challenges and Methodological Considerations in Untargeted Metabolomics Research

Untargeted metabolomics plays a pivotal role in modern biomedical research, enabling the comprehensive study of metabolites to uncover insights into complex biological systems and disease mechanisms [225]. Several thousands of signals can be measured for each sample with mass spectrometry detection combined with separation techniques such as liquid chromatography (LC), gas chromatography (GC), or capillary electrophoresis (CE). However, the field is laden with methodological and technical challenges that influence the reliability and reproducibility of results. While MS offers high sensitivity and broad metabolite coverage, it is prone to variations due to sample handling, instrumentation drift, and ion suppression effects. One of the most significant challenges in metabolomics research is data reproducibility. This issue is often exacerbated by instrument variability, batch effects, and the lack of standardized protocols for sample collection and preparation. Many studies emphasize the importance of quality control (QC) and quality assurance (QA) practices to mitigate these issues. There is still a lack of universally accepted standard operating procedures (SOPs) for untargeted metabolomics, which complicates cross-study comparisons and meta-analyses. Nevertheless, several procedures as already comprehensively discussed by Dudzik et al. [226] or Beger et al. [227] and official initiatives such as the Quality Control Consortium (mQACC) [228] aim to standardize the metabolomics workflow. Another limitation lies in the often insufficient sample sizes and poorly characterized cohorts, which restrict the statistical power and generalizability of findings. Additionally, variability introduced by environmental factors, diet, and microbiome interactions can obscure biologically meaningful signals [225]. Data processing including normalization is a critical step in metabolomics, ensuring that observed variations are biologically relevant rather than technical artefacts [229,230]. The integration of metabolomics with other ‘omics’ technologies, such as genomics, transcriptomics, and proteomics, represents an emerging frontier. This interdisciplinary approach offers a systems-level understanding of biological processes, although differing data structures and analytical workflows still pose significant bioinformatics challenges. However, the constant advancements in the field will improve the reliability of metabolomics data, fostering their application in clinical diagnostics, personalized medicine, and systems biology [230]. By addressing current limitations, untargeted metabolomics has the potential to uncover the molecular underpinnings of health and disease.

## 8. Future Directions

### 8.1. Metabolomics-Based Personalized Approach in Atrial Fibrillation Treatment

Current medications for AF have several limitations as they are mainly symptomatic and do not directly address the underlying pathomechanisms [231]. Available data suggest that some metabolically active drugs, commonly used for other CVDs, not only treat those conditions but also reduce the incidence of AF through their multipotent effects [231,232,233,234,235,236,237]. Upstream therapy, which aims to modify the arrhythmogenic substrate and serve as an adjuvant approach to traditional AF treatment, has been postulated but still requires formal approval and clinical validation [231]. Drugs with anti-inflammatory and antioxidant properties, such as statins, ACE inhibitors (ACEIs), angiotensin receptor blockers (ARBs), and omega-3 fatty acids, are particularly beneficial for patients at risk of fibrotic structural remodelling [231]. Beyond treating common causes of atrial fibrillation, like hypertension, diabetes mellitus, or hypercholesterolemia, they target atrial remodelling and help to re-balance the metabolic status of the patient through anti-inflammatory and antioxidant mechanisms, acting against several substrates that maintain the arrhythmia. However, AF is caused by a variety of pathophysiological processes and its metabolic heterogeneity necessitates tailored therapeutic approaches. Different populations, with diverse disease stages and comorbidities, may require distinct treatment strategies [238]. As AF is characterized by significant variability in metabolic profiles across patients, the challenge lies in identifying phenotypes for accurate risk stratification and providing effective individualized therapy.

### 8.2. Metabolomics for Preventing Cardiogenic Stroke in Atrial Fibrillation

Cardiogenic stroke is the most severe consequence of AF, and is associated with significant mortality and severely decreased quality of life [6]. To date, only one study by Zhang et al. has specifically assessed potential biomarkers for cardiogenic stroke, the one caused by AF [239]. Using machine learning, 12 biomarkers, including six metabolites, were identified to predict stroke in AF patients [239]. The altered pathways included arachidonic acid metabolism, serotonergic synapse, purine metabolism, tyrosine metabolism, and steroid hormone biosynthesis [239]. Potential metabolomic biomarkers identified were N-benzylformamide, glycitin, tiapride, 6-methylthiosine, prostaglandin H1, and prostaglandin F2-alpha metyl ester [239]. These findings require further research for validation and to reduce morbidity. Metabolomic analyses, beyond their role in AF prediction, may also serve for the prevention of its consequences and improving decision-making in clinical practice.

### 8.3. From Metabolomics to Precision Medicine in AF: Integrating Genomics

Metabolomics, as part of —omics sciences, serves as a cornerstone of systems biology, by providing a functional understanding of an organism through the interactions of genes, RNA, proteins, and the environment [24]. This approach allows for a precise assessment of an organism’s current phenotype and enhances the detection of changes in both the proteome and genome. Its advantage over proteomic and transcriptomic analyses lies in its ability to reflect the actual phenotypic state rather than merely indicating potential causes, which are less predictive of future outcomes [24]. However, integrating metabolomics with proteomics, transcriptomics, and genomics data can provide a more comprehensive understanding of AF pathomechanisms. Given that current AF therapies are limited in substrate formation for AF and are often of low efficacy, novel multi-omics strategies such as targeted protein modulation or gene therapy, hold promise for advancing precision medicine [240,241].

A significant percentage of AF cannot be fully explained by traditional risk factors. Available data suggests that AF has a considerable genetic component, evident in both rare familial cases with Mendelian inheritance patterns and the general population [242,243]. However, the clinical utility of genetic findings for risk stratification remains under debate. Mainly ion channel function-related, structural protein-related, and atrial fibrosis-related genetic variations have been found to be important factors in AF vulnerability [242]. Additionally, genome-wide association studies (GWASs) have linked several single-nucleotide polymorphisms (SNPs) to an elevated risk of AF [244].

Metabolomics’ contribution to the current advances in AF research in genomics is substantial [245]. Myocardial tissue metabolism is closely linked to ion channel expression [246,247], suggesting that identifying specific metabolic pathways could uncover genes responsible for AF development. Recent studies of metabolite profiles in AF-patients indicate a possible pathophysiological role of metabolic alterations in AF. However, further work is essential to explain in detail heritability and specific metabolic processes leading to AF. The Cooperative Health Research in South Tyrol (CHRIS) study demonstrated the potential clinical benefit of integrating genomic and metabolomic approaches [107]. For instance, a GWAS identified novel loci, such as SNPs rs745582874 (near the *PBX1* gene) and rs768476991 (within gene *PCCA* gene—Propionyl-CoA carboxylase subunit alpha), which may serve as rare genetic determinants of AF. These findings, alongside elevated lysoPC a C20:3 levels, underline the value of combining genomics and metabolomics in AF research and screening [107]. Translating these discoveries into disease pathways could lead to novel therapeutic strategies [248].

Genomics insights offer significant potential in precision medicine for AF management [248,249]. Approaches such as ablatogenomics, which precisely guides the ablation strategy based on a patient’s genetic profile, could ensure procedures are performed only with a strong likelihood of favourable results, reducing complication rates [249]. For example, a common genetic variant within chromosome 4q25 was found to be a powerful predictor of therapeutic success [249]. Similarly, Shoemaker et al. documented a link between the rs22200733 SNP and an increased risk of atrial tachyarrhythmias after catheter ablation for AF [250]. These findings underscore the promise of omics technologies in enabling personalized medicine, targeting the specific pathophysiology of each patient to optimize outcomes while minimizing adverse events [248,249]. Despite these promising advancements, much remains to be discovered. Current findings require further validation and clinical implementation to realize the full potential of multi-omics approaches in AF management [251].

## 9. Conclusions

Atrial fibrillation, despite its high prevalence, poses a significant healthcare burden nowadays due to unspecific symptoms, far-reaching medical consequences and a lack of appropriate screening tools. Although numerous risk factors have been linked to AF, none are sufficiently specific or sensitive to reliably predict its onset. This highlights the need for novel prognostic methods, such as metabolomics assessment, to improve AF prediction and patient outcomes. Metabolomic analyses show promise as a tool for arrhythmia monitoring; however, current evidence is scarce and findings are often inconsistent, especially considering methodology limitations, i.e., sample sizes or various classification criteria. Further in-depth research is essential to establish more reliable approaches for managing AF, including risk stratification and personalized treatment, and, therefore, reducing cardiovascular mortality.

## Figures and Tables

**Table 1:** Human-based metabolomics studies that report AF-associated findings.

Study	Study Population/Sample Size	Analysed Material	Changes Reported in AF	Main Findings
Mayr, 2008 [91]	Patients (n = 45) undergoing cardiac surgery for nonrheumatic valvular disease or CABG Study group: persistent AF Control group: SR	LAA	↑ beta-hydroxybutyrate, ketogenic amino acids (leucine and tyrosine), and glycine, fumarate, and fumarate/succinate ratio in persistent AF ↑ glycolytic end-products/lipid end-products in POAF after CABG ↓ glucose, beta-hydroxybutyrate, and acetate in POAF after CABG ↓ glucose and creatinine in POAF after valve surgery ↓ glucose/acetate ratio in all POAF patients	- A potential role for ketone bodies in AF and metabolic changes in persistent AF was indicated. - Biomarkers for POAF were proposed based on the metabolomic profile in POAF regarding the type of surgery.
Alonso, 2015 [92]	African Americans (n = 118) from Atherosclerosis Risk in Communities study without AF at baseline	serum	↑ glycolithocholate sulphate and glycocholenate sulphate	- A novel association was found between the incidence of AF and higher levels of two secondary bile acids. - Possible connections between the etiopathogenesis of AF and bile acid metabolism were found.
Ko, 2016 [93]	2458 European ancestry participants from the Framingham Heart Study	plasma	none of the metabolites tested were significantly altered	- The authors recognised several limitations of their study that may explain the inconsistency between results reported in the literature.
Lai, 2018 [94]	AF (n = 49) and non-AF (n = 116) patients undergoing cardiac surgery	LAA and plasma	- seven significantly altered pathways involving LAA metabolism: aminoacyl-tRNA biosynthesis, arginine and proline metabolism, glycine, serine, and threonine metabolism, purine metabolism, taurine and hypotaurine metabolism, pantothenate and CoA biosynthesis, and beta-alanine metabolism - six significantly altered pathways involving plasma sample metabolism: valine, leucine, and isoleucine biosynthesis, glycerophospholipid metabolism, valine, leucine, and isoleucine degradation, pantothenate and CoA biosynthesis, arginine and proline metabolism, and propanoate metabolism	- A total of 24 metabolites in both LAA and plasma reflected metabolic differences between AF and non-AF patients. - Creatinine, D-glutamic acid, choline, hypoxanthine, and niacinamide in LAA and plasma were considered prominent for AF patients. - In plasma samples, the combination of D-glutamic acid, creatinine, and choline was defined as a set of metabolite biomarkers for the recognition of AF and non-AF patients.
Jung, 2018 [95]	182 patients who underwent ECV for persistent AF, 52 healthy controls	plasma	↑ longer saturated FAs (C18:0, C20:0, C22:0) ↓ MUFA and PUFA	- FA alterations in AF patients may be associated with enhanced inflammation, which contributes to disease understanding and allows for future targeted therapy. - The pattern was similar between recurrent and non-recurrent AF.
She, 2018 [96]	AF patients (n = 23) vs. controls (n = 37)	plasma	↓ 4-hydroxy-pyrrolidine-2-carboxylic acid in persistent AF ↑ L-3-aminoisobutyric acid, D-allothreonine, 4-hydroxy-pyrrolidine-2-carboxylic acid, L-lysine, L-valine, L-threonine, L-methionine, L-isoleucine, glycine, L-leucine, and hypotaurine	- Several amino acids showed significant prediction value for AF. - An enhanced AA correlation network further identified AF as a metabolism disorder which gives insight into the pathogenesis of the disease and may allow for targeted future therapy.
Harskamp, 2019 [97]	MURDOCK Cardiovascular Disease Study: AF-free patients (n = 1892)	plasma	↑ medium- and long-chain acylcarnitines and short-chain dicarboxylacylcarnitines	- A total of 233 patients developed AF, risk factors included the elevation in acylcarnitines level, suggesting its predictive value.
Zhou, 2019 [98]	Study group: AF patients (n = 50) Control group: non-AF angiocardiopathy patients (n = 50) with CVD Cohorts matched with age and gender, Chinese—Beijing	plasma	purine metabolic pathway and FA metabolism were perturbed by AF onset	- FA 20:2 and FA 22:4 demonstrate a strong linear correlation with the left atrial area, suggesting their potential as valuable biomarkers for monitoring the progression of AF or for prognosis assessment.
Alonso, 2019 [99]	3922 participants from the ARIC study without AF history	plasma	↑ glycocholenate sulphate, pseudouridine, uridine, and acisoga.	- The study confirmed a prospective link between glycocholenate sulphate, a previously identified secondary bile acid, and the incidence of AF. New metabolites related to nucleoside and polyamine metabolism were identified as potential markers of AF risk.
Yan, 2019 [100]	AF (n = 14), and SR samples as control (n = 12)	plasma	↑ potassium, sodium ion, chitin, benzo[a]pyrene-7,8-dihydrodiol-9,10-oxide, and Celebrex	- Potassium and sodium ions could serve as potential biomarkers for AF. Furthermore, they may offer important insights for early diagnosis and the development of therapeutic targets for AF.
Smith, 2020 [101]	3770 participants in the Malmö Diet and Cancer Study	plasma	↑ medium- and long-chain acylcarnitines, caffeine, and acisoga were associated with an increased risk of AF; beta carotene was associated with a lower risk of AF	- The majority of metabolites linked to incident AF were acylcarnitines, indicating a long-standing dysfunction in carnitine metabolism that predates AF diagnosis and may play a role in its pathogenesis.
Li, 2021 [102]	Prospective study of 391 patients undergoing CABG without AF history	plasma	↑ cholic acid and N-Acetyl-L-aspartate ↓ urocanate, L-allothreonine, homocysteine, and 16-hexadecanol in POAF	- The dysregulation of the PPARα and glutathione metabolism pathways, which are linked to metabolic remodelling and redox imbalance-associated electrical remodelling, may play a critical role in the development of POAF. A total of 96 patients (25%) developed POAF.
Lind, 2021 [103]	Three independent populations with available data on AF onset de novo during 10–12 years on follow-up	plasma	↑ 9-decenoylcarnitine	- 9-Decenoylcarnitine as a novel replicated biomarker for atrial fibrillation was found; however, this acylcarnitine is unlikely to have a causal relationship with the condition.
Li, 2021 [104]	Prospective study of 100 patients with MVD undergoing MVR with chronic AF (n = 30) and SR as a control (n = 26)	plasma	↑ galactose metabolism (lactose, sucrose, galactinol, and raffinose) ↑ unsaturated fatty acids (arachidonic acid, palmitic acid, and linoleic acid)	- Integrated proteomic and metabolomic analyses highlight the significant role of the PPAR signalling pathway in AF. - Other pathways such as the RAAS, galactose metabolism, the biosynthesis of unsaturated fatty acids, and linoleic acid metabolism were implicated in MVD-associated AF.
Hu, 2021 [105]	10 persistent non-valvular AF and 10 valvular AF Control: 10 SR patients	LAA	- 58 ↑ and 49 ↓ regulated metabolites in non-valvular AF, compared to healthy donor hearts; glutathione was the most increased and 20-hydroxy-PGF2a was the most decreased - 47 ↑ and 122 ↓ regulated metabolites in non-valvular AF compared to healthy donor hearts; thymine and thiopental were changed the most significantly	- The enrichment pathways associated with non-valvular AF differ significantly from those of valvular AF. - Specifically, valvular AF shows a strong connection to glycolysis/gluconeogenesis, whereas non-valvular AF is more closely tied to the TCA cycle. This suggests that distinct metabolic processes underlie these two forms of AF, highlighting the importance of tailored therapeutic approaches.
Razquin, 2021 [106]	502 incident AF cases individually matched with ≤3 controls	plasma	↑ quinolinic acid in AF	- Metabolites from the tryptophan kynurenine pathway were found to be prospectively linked to an increased risk of HF and, to a lesser extent, to AF risk.
Emmert, 2021 [107]	110 AF cases out of 10,509 general population individuals in South Tyrol study	plasma	↓ lysoPC a C20:3	- Reduced levels of lysoPCa C20:3 in AF may contribute to AF progression via the activation of the NLRP3 inflammasome.
Zhang, 2021 [108]	A pooled sample of 30 healthy volunteers, a total of 159 samples: - Discovery set (49 with AF and 46 controls) - External validation set (36 with AF and 28 controls)	plasma	↓EtherPC16:1e_20:4, EtherPE 16:1e_20:4, FA 23:0, FA 24:0, FA 25:0, FA 26:0, FA 26:1, LPC 16:0/0:0, LPC 18:2/0:0, LPC 20:4/0:0, LPC 20:5/0:0, LPC-O 18:3, LPE 16:0, LPE 18:2, LPE 20:4, LPE 22:6, PC 15:1_18:2, PC 36:7, PC 38:8, PC 40:8, PE 16:0p_20:4, PE 18:0_18:1, PE 18:0_18:2, PE 18:1_18:2, PE 18:1_20:4, PI 16:0_18:1, PI 16:0_18:2, PI 36:3, PI 16:0_20:4, PI 16:0_22:6, PI 17:0_18:1, PI 18:0_18:2, PI 38:5, SM d40:0, SM d40:1, and SM d42:1	- A combinational biomarker comprising LPC 20:5, FA 23:0, and PI 16:0_18:1 was identified, capable of distinguishing AF patients from control subjects in the discovery set. This finding indicates that the biomarker effectively differentiates AF patients from healthy individuals. Notably, FA 23:0 emerged as a key component in diagnosing AF.
Zuo, 2022 [109]	24 patients with a history of AF and 24 non-AF controls	faecal samples	↓ SCFA (acetic, butyric, and propionic), with a declining trend from paroxysmal to persistent AF	- Findings suggest that activating atrial GPR43 through SCFAs represents a promising therapeutic approach for AF.
Goni, 2022 [110]	Incident AF cases (n = 509), controls without incident AF (n = 547), PREDIMED trial	plasma	↓ arginine ↑ N1-acetyl-spermidine	- Metabolites associated with arginine catabolism may play a role in AF and HF.
Lu, 2022 [111]	363 patients, general population; groups: - Healthy control - Suspected AF - First diagnosed AF - Paroxysmal AF - Persistent AF - After stroke	plasma	↓ oleic acid ↑ D-glutamic acid and uric acid comparing AF vs. healthy ↑ 2-ketoglutaric acid, lactose, and 2-hydroxybutyric acid ↓ glycerol-2-phosphate, O-phosphoryl-ethanolamine, and LysoPC (20:0/0:0) in All-Afs group	- In comparison to the control group, the All-AFs group combined with Car-AF and Sus-AF groups exhibited similar metabolic pathways, including those involved in alanine, aspartate, and glutamate metabolism, as well as D-glutamine and D-glutamate metabolism. - Certain compounds, such as 3-hydroxybutyric acid, homocysteine, aminomalonic acid, and uridine, were specifically detected in Car-AF (compared to All-AFs). This suggests a potential link between these metabolites and the development of cardiogenic cerebral embolism resulting from AF.
Liu, 2023 [112]	10 AF patients undergoing surgical ablation of AF without cardiopulmonary bypass with non-valvular persistent AF patients Control group: 10 matched samples from healthy donors for transplantation	LAA	113 significantly ↑ and 10 significantly ↓ metabolites, alterations of raffinose, adenine, and D-Mannitol in AF	- In GSVA, the expression levels of GPD2, PLEC, and SYNM were shown to be linked to various metabolic processes associated with mitochondrial function (e.g., lipid metabolism and AMP-activated protein kinase signalling) as well as cardiac structural and electrical remodelling. These mechanisms are crucial for the onset and persistence of AF. - New insights into the progression of AF, particularly highlighting the role of mitochondrial metabolic reprogramming and identifying several mitochondrial function-related genes as potential novel targets for AF treatment.
Yang, 2023 [113]	Chinese population, two cohorts of patients after isolated CABG without AF history, n1 = 306, n2 = 207	pericardial fluid and plasma	↑ aceglutamide, ornithine, methionine, and arginine in predicting POAF	- The multi-metabolite model demonstrated excellent predictive performance in both the discovery and validation sets. Blood-based metabolite detection could be a valuable tool for predicting and preventing POAF.
Li, 2023 [114]	2059 adults in the FHS cohort	plasma	↑ glycerol, cholesterol ester 16:1, and phosphatidylcholine 32:1	- Metabolomics offers valuable insights into CVD and AF development.
Toledo, 2023 [115]	512 incident AF cases and 735 controls	plasma	↑ PC plasmalogens and PE plasmalogens, palmitylo-EA, cholesterol, CE 16:0, PC 36:4;O, and TG 53:3.	- No interaction with the dietary intervention was observed. A multi-lipid score, predominantly composed of plasmalogens, was linked to a higher risk of AF.
Zhang, 2024 [116]	54 pts, general population (36 AF and 18 control): - Persistent AF - AFPA - SR	plasma	Paroxysmal AF: ↑ arachidonic acid, glycolic acid, and L-serine Persistent AF: ↑ glycolic acid, L-serine, and palmitedaic acid.	- Differential metabolites were validated with high sensitivity and specificity, making them potential biomarkers for diagnosing and monitoring AF progression.
Huang, 2024 [117]	30 persistent AF patients and 30 controls	plasma	↑ lysine, tyrosine, glutamic acid, methionine, and isoleucine, and ↓ glycine in persistent AF	- Alterations in AA levels may serve as biomarkers for AF, and restoring their balance could offer potential benefits in managing age-related AF.
Soulat-Dufour, 2024 [47]	85 consecutive patients hospitalized for AF with restoration of sinus rhythm at 6 months	plasma	↑ kynurenine, tryptophan, and urea/creatinine ↓ arginine and methionine/methionine sulfoxide in AF	- AF can be predicted through a combination of clinical, biological, metabolomic, and echocardiographic variables assessed at admission.

AA—amino acids, AMP—adenosine monophosphate, AF—atrial fibrillation, AFPA—paroxysmal atrial fibrillation, ARIC—Atherosclerosis Risk in Communities, CABG—coronary artery by-pass grafting, Car-AF—cardiogenic ischemic stroke atrial fibrillation, CoA—coenzyme A, CE—hexadecanoate, CVD—cardiovascular disease, ECV—electrical cardioversion, etherPC—ether-linked phosphatidylcholine, etherPE—ether-linked phosphatidylethanolamine, FA—fatty acid, FHS—Framingham Heart Study, GPD2—glycerol-3-phosphate dehydrogenase 2, GPR43—G-protein–coupled receptor 43, GSVA—Gene Set Variation Analysis, HF—heart failure, hydroxy-PGF2a—hydroxy-postaglandin F2α, LAA—left atrial appendage, LPC—lysophosphatidylcholine, LPC-O — ether-linked lysophosphatidylcholine, LPE—lysophosphatidylethanolamine, MedDiet—Mediterranean diet, MUDROCK—Measurement to Understand Reclassification of Disease Of Cabarrus and Kannapolis, MUFA—monounsaturated fatty acids, MVD—mitral valve disease, MVR—mitral valve repair, NLPR3—nucleotide-binding domain, leucine-rich–containing family, pyrin domain–containing-3, NMR—nuclear magnetic resonance spectroscopy, palmitoyl-EA—palmitoylethanolamide, PC—phosphatidylcholines, PE—phosphatidylethanolamine, PGF2α—prostaglandin F2α, PI—phosphatidylinositol, PLEC—plectin, POAF—postoperative atrial fibrillation, PPARα—peroxisome proliferator-activated receptor alpha, PUFA—polyunsaturated fatty acids, RAAS—renin–angiotensin–aldosterone system, SCFA—short-chain fatty acids, SR—sinus rhythm, Sus-AF—suspected atrial fibrillation, SM—sphingomyelin, SYNM—synemin, TCA—tricarboxylic acid, TG—triacylglycerol, tRNA—transfer ribonucleic acid, ↑ rise in level, ↓ decrease in level.
